# Evaluating Uncertainty in Signaling Networks Using Logical Modeling

**DOI:** 10.3389/fphys.2018.01335

**Published:** 2018-10-09

**Authors:** Kirsten Thobe, Christina Kuznia, Christine Sers, Heike Siebert

**Affiliations:** ^1^Group for Discrete Biomathematics, Department for Mathematics and Computer Science, Freie Universität Berlin, Berlin, Germany; ^2^Group for Mathematical Modelling of Cellular Processes, Max-Delbrück Center for Molecular Medicine, Berlin, Germany; ^3^Laboratory of Molecular Tumor Pathology, Institute of Pathology, Charité Universitätsmedizin Berlin, Berlin, Germany; ^4^Laboratory of Bioorganic Synthesis, Department of Chemistry, Humboldt-Universität zu Berlin, Berlin, Germany

**Keywords:** systems biology, logical modeling, model checking, constraint based modeling, signaling pathways

## Abstract

Systems biology studies the structure and dynamics of biological systems using mathematical approaches. Bottom-up approaches create models from prior knowledge but usually cannot cope with uncertainty, whereas top-down approaches infer models directly from data using statistical methods but mostly neglect valuable known information from former studies. Here, we want to present a workflow that includes prior knowledge while allowing for uncertainty in the modeling process. We build not one but all possible models that arise from the uncertainty using logical modeling and subsequently filter for those models in agreement with data in a top-down manner. This approach enables us to investigate new and more complex biological research questions, however, the encoding in such a framework is often not obvious and thus not easily accessible for researcher from life sciences. To mitigate this problem, we formulate a pipeline with specific templates to address some research questions common in signaling network analysis. To illustrate the potential of this approach, we applied the pipeline to growth factor signaling processes in two renal cancer cell lines. These two cell lines originate from similar tissue, but surprisingly showed a very different behavior toward the cancer drug Sorafenib. Thus our aim was to explore differences between these cell lines regarding three sources of uncertainty in one analysis: possible targets of Sorafenib, crosstalk between involved pathways, and the effect of a mutation in mammalian target of Rapamycin (mTOR) in one of the cell lines. We were able to show that the model pools from the cell lines are disjoint, thus the discrepancies in behavior originate from differences in the cellular wiring. Also the mutation in mTOR is not affecting its activity in the pathway. The results on Sorafenib, while not fully clarifying the mechanisms involved, illustrate the potential of this analysis for generating new hypotheses.

## 1. Introduction

**Figure 1:**
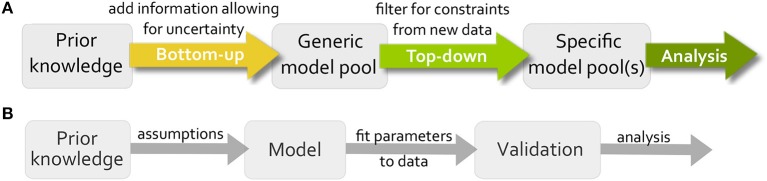
Workflow for our modeling approach in comparison to traditional modeling process. **(A)** First a generic model pool is created from all available information including uncertainty. Then, the pool is filtered for data to find specific subpools, which can be analyzed for new information. **(B)** The traditional workflow creates one model based on assumption, estimates parameter values by fitting the model to data and validates the model on additional data.

Logical modeling has been shown to be a powerful tool for representing and analyzing biological systems (Saez-Rodriguez et al., 2007; Wang et al., 2012; Grieco et al., 2013). The main advantage in comparison to the standard modeling formalism in systems biology, Ordinary Differential Equations (ODE) modeling, is the low number of parameters, therefore logical models are mainly used to build large models that would be too complex for ODEs (Abou-Jaoudé et al., 2016). These models are usually built in a bottom-up manner, which means all available information about the system is gathered and validated on new data (Figure 1B). A main issue when building these models is that uncertainty cannot be included into the model, e.g., if an influence between two components is controversial in the literature. Since only one model is created, the modeler needs to make an assumption neglecting the uncertain information. A second popular strategy for modeling is to use a top-down approach where the model is inferred directly from data, but here prior knowledge about the system is neglected (De Smet and Marchal, 2010).

As a consequence, alternative approaches have become more popular, where uncertainty is included into the modeling process by either building more than one model or by adapting the model through training. For exploring a wide range of models that is able to show a certain dynamical behavior, called family or pool of models, there are different methods available. The group of Saez-Rodriguez et al. developed a software *CellNOptR* to train a candidate model to data, accounting for topological uncertainties (Terfve et al., 2012). The output is a family of models selected for an optimality criterion, but cannot guarantee completeness due to stochastic search. The software *caspo* by the group of Siegel et al. uses Answer Set Programming to infer a family of logical models from experimental data based on optimization, where a tolerance accounts for experimental noise. The resulting family of models then represents all optimal models that reproduce the data and the software provides several analysis tools to explore properties of the models, such as classification for input/output behavior or experimental design (Videla et al., 2017). A similar method using time series data for inferring a model pool showed to be more precise than *caspo* (Ostrowski et al., 2016). A different approach was developed by our group, where uncertainty in parameters of the model, such as an uncertain sign of an edge, is encoded into the model definition (Klarner et al., 2012) and all possible models that arise from this uncertainty are enumerated. Subsequently the models are tested for satisfiability for data without an optimality criterion (Figure 1A), which was implemented using efficient formal verification techniques in *Tremppi* (Streck et al., 2015) and in *TomClass* (Klarner, 2014). Even though we employ the software from our group for the analysis in this paper, one could apply different software along the pipeline for building the model pool or analyzing it.

While computing model pools and testing them for data sets is computationally challenging, the analysis of potentially hundreds or thousands of models is not straight-forward in terms of the biological interpretation. Thus we propose a hypothesis-driven approach for specific biological questions, where the use of model pools allows us to test multiple hypotheses at the same time and analyze their interdependencies. Mathematical models are artificial constructs used to help understanding biological processes. In order to receive meaningful results from a modeling study, the biology needs to be transferred into mathematics and the results need to be interpreted from a biological perspective. In this paper, we address this task of incorporating biological information into the formalism by expanding the workflow in Figure 1 to a four-step pipeline. At first, the process of bottom-up model building formalizes the biological phenomena into a prior knowledge network, which we call *system initialization*. Here, the regulatory graph and the logical equations are derived from literature information. Then, the *objective formalization* includes the aim of the study into the model setup, e.g., by adding extra components or edges. After generating the model pool, the top-down filtering process uses biological data that is not restricted to be of a specific type such as steady-state or input-output behavior. However, it requires a *data formalization* step. Finally, the *pool analysis* examines the specific pool for new biological insight.

In previous work, we presented parts of this pipeline, i.e. the objective of investigating crosstalk between two signaling pathways in Thobe et al. (2014), as well as challenges for data discretization and analysis in Streck et al. (2015) in context of a specific software. Here, we generalize and expand this pipeline by two additional objectives and analysis methods. Especially in the context of signaling processes in cancer cells, the identification of driver mutations is of great interest (Bozic et al., 2010), thus one aim of our framework is to identify driver mutations by a change in the logical function. The second aim presented is testing the effect of drugs by introducing them as new inputs to the system. Analyzing pools containing possibly hundreds or thousands of models is challenging. Here, we show a classification analysis to structure the resulting models toward interesting features, as well as extracting minimal mechanisms for a more detailed view on the models.

We apply this pipeline to model two central signaling processes involved in cancer, the mitogen-activated protein kinase (MAPK) cascade and the mTOR pathway (Shaw and Cantley, 2006; Saini et al., 2013), in two renal cancer cell (RCC) lines. Both cell lines were treated with the Raf-inhibitor Sorafenib yet displayed a differential response in terms of apoptosis induction (Kuznia, 2015). We hypothesized that the difference between these cell lines might be caused by distinct wiring of MAPK and mTOR signaling, which were shown to be connected via crosstalk (Mendoza et al., 2011; Aksamitiene et al., 2012). A rich dataset of time series measurement of key components in both pathways was generated using a high-throughput method, which was the foundation for the complex analysis presented in this study.

This paper is organized as follows. The Methods section first gives a brief introduction on the logical modeling framework and a detailed description on the pipeline we developed. In the Results section, the application on a signaling network is demonstrated, where first the model building process with the corresponding biological background is given, the data processing procedure is described and the results of the analysis are presented. Additionally, the biological interpretation is discussed and future experiments are suggested to wrap up the application section. Finally, the Discussion section exploits advantages and shortcomings of the method showing potential future extensions.

## 2. Methods

### 2.1. Theoretical background

The formalization of logical modeling for biological systems was introduced by Kauffman (1969) and further refined by Thomas (1991), which is the base for our work. However, we expanded this formalism to incorporate uncertain information leading to model pools (Klarner, 2014; Thobe et al., 2014, 2017; Streck, 2015; Streck et al., 2015).

#### 2.1.1. Logical modeling

The topology of a biological system is defined as a directed graph R=(V,E,l), called *interaction graph* (IG), where the nodes *V* = {1, …, *n*} represent the *components* of the system that are connected by edges *e*∈*E*⊆*V*×*V* called *interactions*, which represent a regulation of one component by another. The components adapt discrete values, called *activity levels*, and we consider *Boolean networks* (BN) with two levels assigned to each component *B* = {0, 1}, where 0 means inactive and 1 stands for active. By assigning activity levels to every component of the network, the *state* of the system *s* is defined by *s*:*V* → {0, 1}, ∀*v*∈*V*:*s*(*v*)∈*B*. Here, the notation of a state is specified as a sequence in the order of *V*. In our approach, we add information about the nature of a regulation to each interaction using edge labels *l*:*E* → {+, −, ¬+, ¬−} (adapted from Klarner, 2014). In application, the labels {+, −} are assigned to edges that represent well-known information, e.g., textbook knowledge, and are therefore required to be present in every model, which we call *essential*. In contrast, the labels {¬+, ¬−} are assigned to interactions that carry uncertainty, i.e., we not sure whether this interaction is present or not, which we call *optional*. However, we assume that the sign of an edge is known and exclude edges with unknown or ambivalent sign due to complexity.

**Figure 2:**
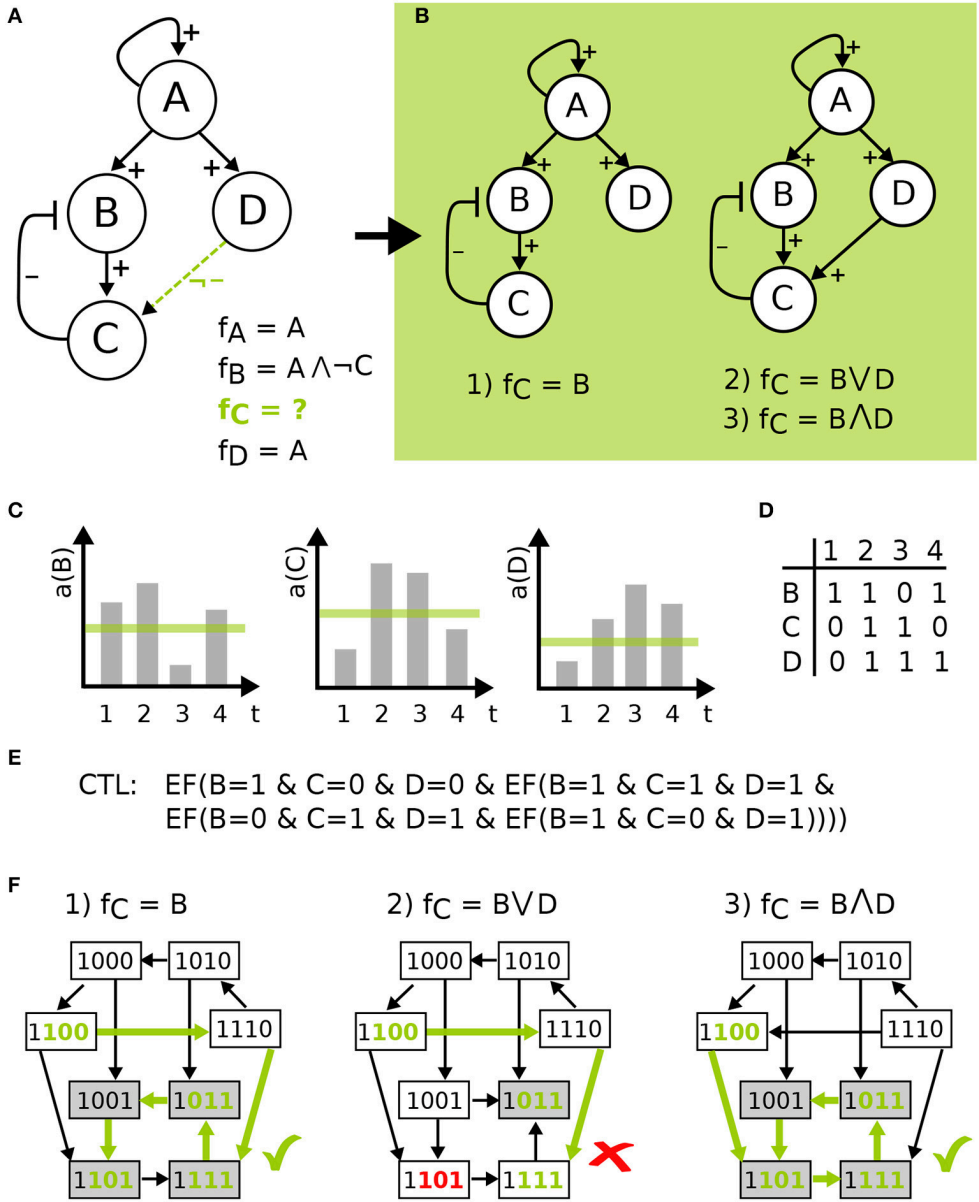
Model definition to model checking visualized on a toy example. **(A)** IG with four components, edge labels and the corresponding functions resulting in model pool of size three in **(B)**. **(C)** shows time-series measurements for some activity *a* of B, C, and D, which is discretized by a threshold shown in green. The table in **(D)** gives the discretized data for the four time points, which are encoded as CTL formulas in **(E)**, where EF(X) is a CTL operator *exists finally*. This states that on some path from an initial state the X holds true at some point. STGs in **(F)** of the three models in the pool show the process of model checking for the CTL formula indicated by green states and edges, where the second model is not in agreement with the data.

Having defined the wiring of the network, the regulation of a component by its predecessors is defined by a logical function. The conditions describing when a component becomes active can be expressed using the logical operators ∨ (OR), ∧ (AND), and ¬ (NOT) in a formula *f*_*i*_ for every component *v*∈*V* consistent with the edge labels. This means that variables *j* are literals in *f* for component *i*, if *j*→*i* is a possible edge. Then a positive edge label has to cause an increasing value in the target component at some point, whereas a negative edge label has to cause a decrease. For optional edges, the increase or decrease can occur or that value is constant. However, in case the regulation of a component is uncertain of a component has optional incoming edges more than one model can be build from the available information. Then the set or all logical equations that are consistent with the edge labels are created and form the so-called *model pool*. An example is given in Figure 2.

#### 2.1.2. Dynamical behavior and model checking

In order to compare biological measurements with the dynamic behavior of the models, we need to define the transition from one state to another to generate the systems behavior over discrete time steps. For this aim, different update strategies have been developed, where some make assumptions on the timing of events, e.g., in synchronous update all components change in one transition, and others restrict the ordering of events, e.g., stochastic updates randomly update a component. Here, we employ asynchronous update, which is the least restrictive strategy at the cost of being computationally expensive (Thomas, 1991). In this strategy only one component can change its value per transition step, which means for *f*_*v*_(*s*) = ¬*s*_*v*_ for a state *s* = (*s*_1_, …, *s*_*v*_, …, *s*_*n*_) denote with ${\bar{s}}^{v}=\left(s_{1},\ldots ,\neg s_{v},\ldots ,s_{n}\right)$ the state which differs from *s* in the value of the component *v*. If no component changes *f*(*s*) = *s* a steady-state of the system is reached. This update schedule produces every possible trajectory emerging from a state, thus the dynamics are non-deterministic which can be visualized in the so-called *state transition graph* (STG). Here, the states are the nodes of the graph and the transitions are edges.

After building the model pool from the available information, we want to filter those model that are in agreement with observed experimental data. Depending on the utilized software, either the data can be implemented as continuous values (e.g., Terfve et al., 2012) or needs to be discretized. Here, we want to describe two different kinds of biological data: time-series measurements and steady state observations. For this aim, we use *temporal logics* that are able to describe an ordering or a sequence of events in time, where *computation tree logic* (CTL) can cope with non-deterministic sequences (Clarke et al., 1986) and are therefore suited to explore the STG. For time-series measurements we encode a series of states that should exist at some point in the future and for steady states we encode the state of a component(s) that should hold for every state in the future. These formulas are then tested on the STG of the models using *model checking*. This process can be computationally expensive, since the state space exponentially increases with the number of components, also the number of models can quickly add up to thousands of models. For this reason, an efficient model checking software should be employed, e.g., Tremppi (Streck et al., 2015) and TomClass (Klarner, 2014).

##### 2.1.2.1. Toy example

In Figure 2, model definition to model checking is visualized for a toy example. Here, an IG with four components is given, where the regulation of components A, B, and D is known, indicated by the edge labels and the corresponding functions. Component C has an uncertain regulation by component D, therefore the edge is labeled as not inhibiting and the function for C is undefined (Figure 2A). The resulting model pool then contains three different models that arise from the edge label. The process of temporal encoding of data is shown for time-series measurements, which is discretized by a threshold and encoded as CTL formulas, where the CTL operator *exists finally* is used. This operator states that the measurements must lie on one path in the ordering of the measurements in the STG, where there is no restriction made on how many states are visited in between the measurements. The CTL language offers more operators that could be employed depending on the type of data (Klarner et al., 2012), e.g., reflecting that the measurement frequency was so high that one can assume that all qualitative activity changes of each component have been captured. However, in this paper we used the most conservative form. Finally, the process of model checking is visualized in Figure 2F showing that one model is not in agreement with the data.

### 2.2. Pipeline for modeling uncertain systems

**Figure 3:**
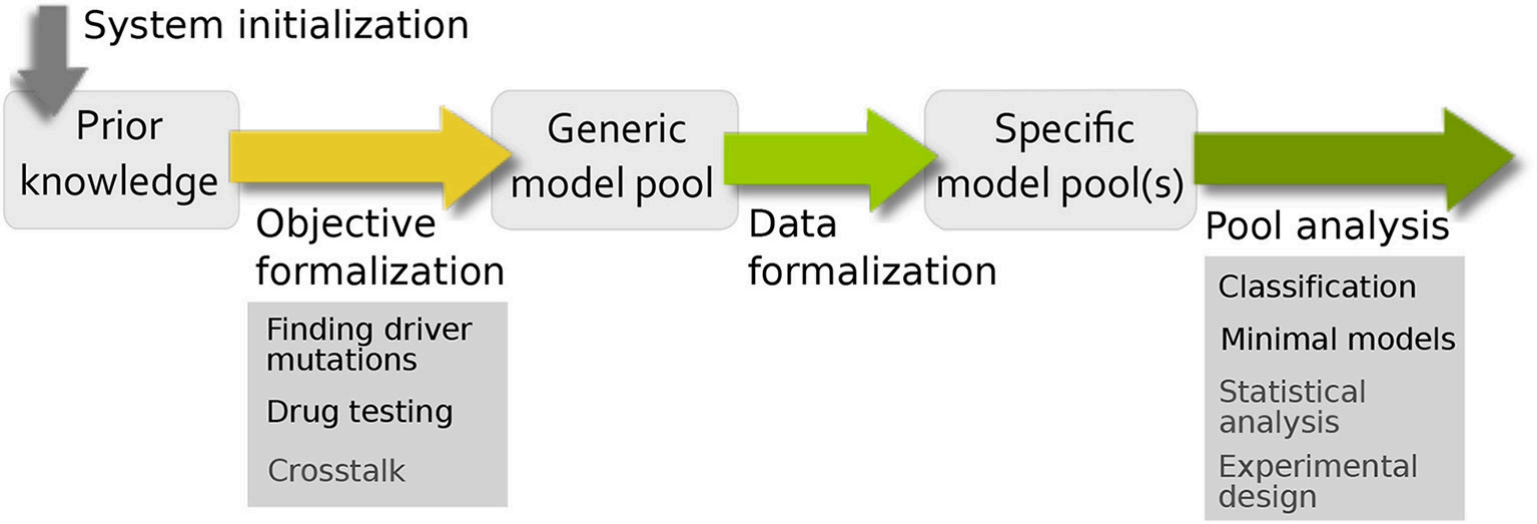
Pipeline for evaluating uncertainty in biological systems. For building the prior knowledge network and defining the uncertainties of the system, the system initialization and objective formalization is necessary. The filtering process from the generic model pool to the specific model pool requires data formalization and the interpretation of the final pool is done by pool analysis.

Based on the very general workflow in Figure 1, we want to formalize in more depth how specific biological questions can be addressed using model pools (Figure 3). In comparison to the traditional workflow for building and analyzing one model, there are similarities and differences. The main difference is that the standard bottom-up approach creates one model to test a single hypothesis, which is validated using (formalized) data and subsequently analyzed. Using model pools, the system initialization and data formalization remains the same, but we can test multiple hypothesis at the same time. This leads to a higher complexity in both the formulation of the aim of the study and the analysis of the model pool for biological information. To this end, the workflow has been adopted and specified to address common analysis themes for signaling networks. The resulting pipeline, shown in Figure 3, contains four steps: system initialization, objective formalization, data formalization, and pool analysis.

In the following, we provide a detailed formal description for the objective formalization and pool analysis. We assume that for the system initialization, available information is gathered and classified according to the theory presented in Section 2.1 for components, edges, and edge labels. Moreover, insight on regulations of components can be included by defining logical functions. This first step results in the *prior knowledge network* (PKN), which forms the starting point for our analysis (Saez-Rodriguez et al., 2011).

#### 2.2.1. Objective formalization

In the second step of our pipeline, we want to include the objective of the analysis into the PKN. In a simple setup, this could mean adding optional edges as hypotheses, but there are also more complex aims that require changes in the PKN. The first objective we identified, was to examine crosstalk between two pathways while preserving the dynamical properties of each pathway, presented in Thobe et al. (2014). Here, we want to present two different objectives: Finding driver mutations and drug testing.

##### 2.2.1.1. Finding driver mutations

Cancer cells often accumulate mutations that are distinguished as either driver or passenger mutations. A lot of effort has been made to identify the driver mutations, since they are assumed to be a major cause for cancerous behavior (Greenman et al., 2007; Bozic et al., 2010). This abnormal behavior is due to the fact that the mutations affect the protein they are encoding in quality, changed sequence of the protein, or quantity, such as overexpression or knock-out of a gene. These effects cause changes in the regulatory network leading to an insensitivity of the component from its regulators, e.g., constantly active receptors. We aim to identify these changes in the regulation of a component in our approach, which we were able to confirm in previous work (Streck et al., 2016).

We account for mutations in components of a model with uncertain effect by setting the respective incoming and outgoing edges to optional even if these connections are textbook knowledge. In case more detailed information on the effect of the mutation is available, only a subset of edges can be set to optional. Formally, the network R=(V,E,l) is defined as in Section 2.1, where the set of mutated components is given by *V*^*m*^⊆*V* with edges *E*^*m*^ = {(*u, v*)∈*E*|*v*∈*V*^*m*^∨*u*∈*V*^*m*^}. The labeling *l*^*m*^ of edges in *E*^*m*^ is set to:

$$l^{m}(u,v)=\{\begin{array}{cc} \neg +\; & \text{if}(u,v)\in E^{m}\text{and}l(u,v)=-\\ \neg - & \text{if}(u,v)\in E^{m}\text{and}l(u,v)=+ \end{array}.$$

Thus, the affected edges are allowed to either stay the same or lose their function in the resulting model pool. If in the specific pool an incoming edge is not observable in any model, the mutated component becomes independent from its inputs and the function of the component can either be set to 0 or 1 indicating a loss-of-function or constitutively active mutation, respectively. A lost outgoing edge of the mutated component can indicate that the mutation affected the protein structure which can result in a dysfunctional protein. However, we do not account for gain-of-function mutations in this set-up, since this would require to add new edges to the model or change the sign of an edge. This would strongly increase the complexity of the study and should be addressed only based on suggestive data in a case by case way, which is no fit for this general set-up.

##### 2.2.1.2. Drug testing

This objective aims to test qualitative effects of drugs on pools of models, without knowledge of the “true” network. Especially in cancer research, combinatorial therapies have become increasingly popular to enhance efficiency and overcome resistances (Ho et al., 2012; Manchado et al., 2016). Since we cannot represent concentrations or generally quantitative effects, the questions we want to address can be formulated as: where do we have to interrupt the signaling process to achieve a certain outcome. A similar study was done by Klinger et al. where they predicted the model structure and treatment quantitatively, however, the predictions resulting from the study were qualitative nature (Klinger et al., 2013).

For this approach, we introduce drugs as new components to the PKN and connect them with an inhibitory edge to their target, since they are supposed to suppress the activity of their target. For the network ${ℛ}^{\prime }=(V^{\prime },E^{\prime },l^{\prime })$ with *f*′ as given logical equations, an extended set of components *V* is given by *V*′∪*V*^*D*^ where *v*^*D*^∈*V*^*D*^ is a set of drug components. The interactions of the network are given by *E* = *E*′∪*E*^*D*^ where new edges *E*^*D*^ are added, which contain an edge for self-activation for each new component to create the drug as input and an inhibitory edge from *v*^*D*^ to its target *u*, since the drug suppresses the activity of its target. Similarly, the set of labels is composed of the labels of the original network and the additional labels for the drug components, where known interactions are labeled with an essential label and uncertain effects with an optional label.

We can also include available information about the drug's mode of action into the logical function of its target. Usually drugs are selected to have a dominant influence on their target, for example through binding or modification it fails to interact with its former regulators. In case the logical equation of a drug target is known, we can directly translate this dominant effect on the target *u* in a new logical equation:

$$\begin{array}{l} f_{u}=f_{u}^{\prime }\wedge \neg v_{D}. \end{array}$$

However, if detailed information about the biochemical properties of the drug on the target and other regulators is missing, the logical equation of the target is not defined and all possible regulations are generated in the pool.

#### 2.2.2. Pool analysis

After building the generic model pool from the PKN, this pool of models gets reduced for those models that are valid for data. Depending on the software, the data needs to be processed to apply it to logical models usually by discretization (Dimitrova et al., 2010; Gallo et al., 2015). As a result, we receive one or more specific model pools that need to be analyzed. For this aim, different kinds of analysis tools can be employed depending on the aim and the size of the resulting model pool such as statistical analysis (Thobe et al., 2014; Streck et al., 2016) or optimization (Terfve et al., 2012; Videla et al., 2017). Here, we want to present an analysis approach that allows a closer look at classes of models as well as single models.

##### 2.2.2.1. Classification

Depending on the study, this pipeline can lead to specific model pools that contain too many models to analyze them by hand. This analysis step aims to get an intuition for commonalities or differences of models within the model pool, with respect to properties of interest. For this goal, properties such as validity for data or presence of an optional edge can be annotated to each model by e.g., using a database. Then, we can group sets of models into classes and compare them according to these properties to find difference between sets of models, for example we could observe that two optional edges are present in the model pool but occur mutually exclusive.

Here, the model pool is stored in a database and SQL queries are used to classify the models. For the queries, properties or a list of properties can be used as a classifier and are defined in the parameter Classes. Also we can restrict the pool to a subpool using the parameter Restriction, where we can select models for their property, e.g., only including all models that carry an optional edge. Mathematically, the analysis finds subsets of models that have a non-empty intersection and computes the cardinalities of these sets (Klarner, 2014). For this aim, an SQL query is generated using statements of the form:

$$\begin{array}{l} \text{SELECT DISTINCT Classes FROM models WHERE Restriction}, \end{array}$$

where SELECT DISTINCT computes all combinations of labels, i.e., subsets, of the selected Classes in the database models, possibly restricted using WHERE. Additionally, COUNT is used to determine the cardinality of each subset, i.e., the number of models in a class later denoted as size of a class. It is possible that classes are empty if there exists no model in the pool with a particular label combination.

##### 2.2.2.2. Minimal models

While the classification gives a broad overview on the model pool, we also want to look at single models in the pool. The selection of models can be motivated by the objective and the biological background, by the classification analysis or by general criteria such as minimality. The criterion minimal can be interpreted in different ways: structural minimality in terms of number of edges, functional minimality in terms of shortest logical equations, or models that require least number of transitions to fulfill data. Each minimality can be interesting to regard separately or in combination. Structural minimality is a common biological assumption, where the system is assumed to have evolved in an energetically optimal way and is therefore sparse. Along with the number of interaction partners, the complexity of the regulation formulated as logical function can be assumed to be rather simple. Previous studies often used fixed rules for creating these functions, such as activation- inhibition function (Martin et al., 2007), or optimized for short logical function (Videla et al., 2017).

Technically, this analysis counteracts the problem of overfitting. In general, the more degrees of freedom are available to a system, the easier is can produce various dynamics, thus our method has a bias toward building dense models. It is therefore beneficial to identify minimal structures or functions.

##### 2.2.2.3. Interpretation of analysis results

Finally, the results from the analysis need to be transferred and interpreted to gain biological insight, which is not straight forward. Since the models are qualitative, the level of abstraction is high and the fact that we are looking at pools of models increases the complexity. However, by specifying clear objectives and predefined analysis options, the pipeline guides the modeling process and can deliver valuable information for experimental design or further modeling steps (Streck et al., 2015; Thobe et al., 2017).

## 3. Results

### 3.1. Application on growth factor signaling in renal cancer cells

After presenting a pipeline to build model pools for different objectives and analysis options, we wanted to apply this pipeline to model growth factor signaling in two renal cancer cell lines, MZ1851RC and MZ1257RC. Motivation for this study was an observation that cell line MZ1851RC showed apoptosis after being treated with the drug Sorafenib while MZ1257RC seemed to be resistant (Kuznia, 2015). Sorafenib was developed to inhibit pathways controlling proliferation and cell survival and was shown to have anti tumor activity in colon, breast, and non-small lung cancer (Wilhelm et al., 2004; Gadaleta-Caldarola et al., 2015). The multikinase inhibitor Sorafenib was designed to suppress activity of Raf kinases in the MAPK pathway (Liu et al., 2006), however, it also affects a wide variety of receptor tyrosine kinases (RTKs) (Wilhelm et al., 2004). Very recently, Sorafenib was shown to inhibit the IGFR *in vitro* (Yaktapour et al., 2013), which indicates that Raf comprises an uncertain drug target in the renal cancer cell lines tested with our approach.

**Figure 4:**
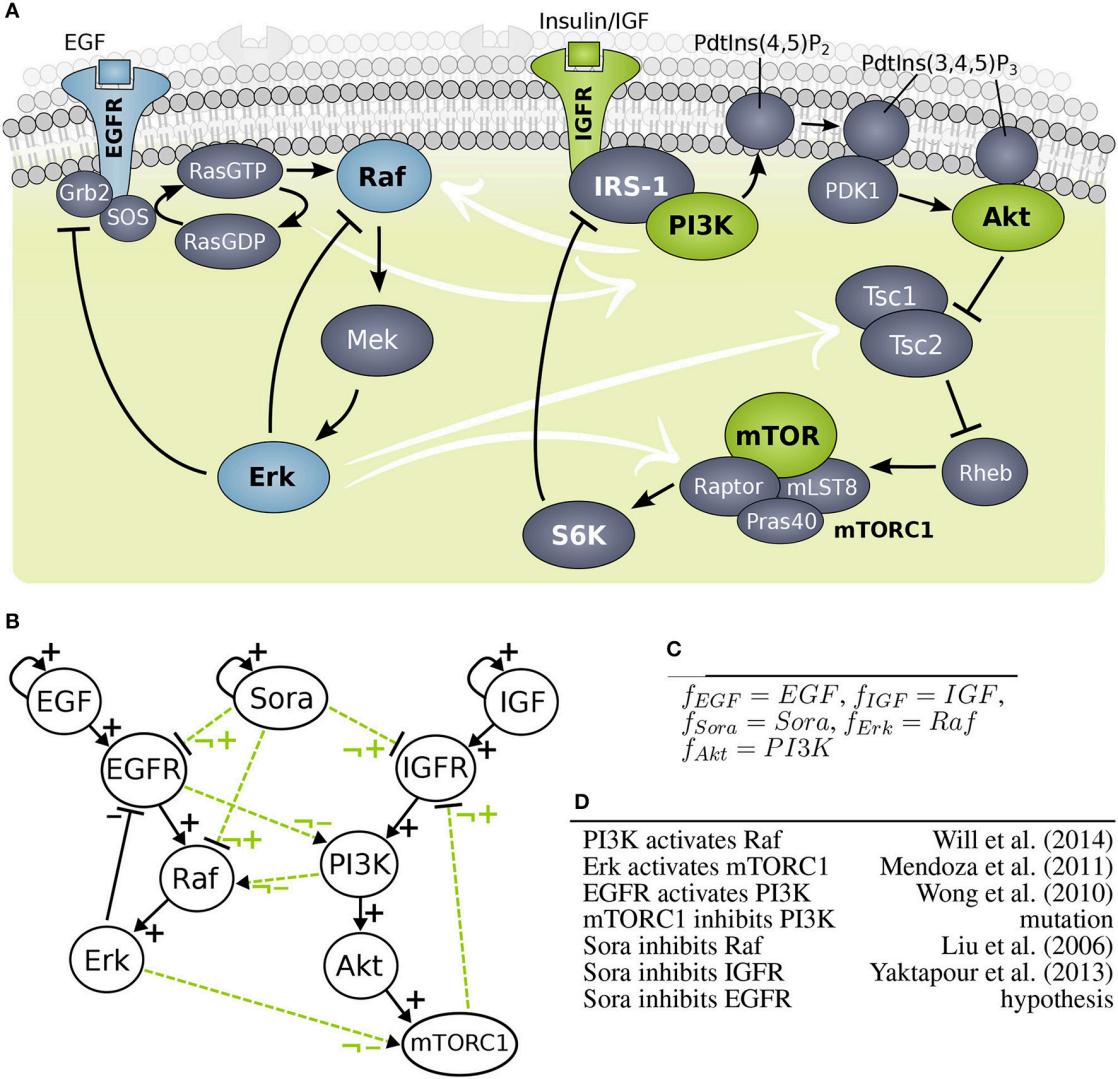
Model building of growth factor signaling processes **(A)** Scheme of MAPK cascade and PI3K signaling. **(B)** Interaction graph of the MAPK (left hand side) and PI3K (right hand side) model marked with solid lines and optional influence of Sorafenib and crosstalk marked with dashed lines. **(C)** Predefined logical rules for regulations of components without optional incoming edges. **(D)** List of optional edges added to the network with references.

A second uncertainty was introduced by a mutation in the component mTOR in cell line MZ1851RC, but the effect of this mutation is unknown (Kuznia, 2015). A third uncertainty was caused by crosstalk between the MAPK pathway and PI3K signaling (Figure 4A), which was shown to compensate drugging of one of the pathways (Mendoza et al., 2011; Aksamitiene et al., 2012). Thus, the overall aim of this study was to clarify if the deviating behaviors are caused by differences the cellular wiring, which effect the mutation has and which targets Sorafenib is affecting.

### 3.2. Objective formalization

The objective of this study splits into three different aims: investigating crosstalk between MAPK and PI3K pathways, finding the target for Sorafenib, and clarifying the effect of the mutation in mTOR. The PKN was extracted from literature, where the MAPK model was based on work by Kholodenko (2000) and the PI3K model was adapted from Courtney et al. (2010), also it is an adaption from a previous study (Thobe et al., 2014). For investigating the wiring between MAPK and PI3K pathway, candidate crosstalks were added. In detail, strongly activated MAPK signaling was found to cross-activate PI3K signaling, i.e., Erk was observed to phosphorylate Tsc2 suppressing it and Erk was also shown to phosphorylate Raptor, where both crosstalks activate mTORC1 signaling similarly to Akt (Roux et al., 2004; Winter et al., 2011). For simplicity, we summarized this effect to one crosstalk. Moreover, a cross-activation of EGFR on PI3K through Ras was shown, which is downstream of EGFR and upstream of Raf (Wong et al., 2010). A study of Will et al. found that PI3K inhibition, but not Akt inhibition, causes rapid decrease in wild type Ras activity and in Raf/Mek/Erk signaling concluding that PI3K cross-activates the MAPK cascade (Will et al., 2014). For the PKN, the crosstalk edges were labeled as optional edges and the edges within a pathway were assumed to be essential, shown in Figure 4B.

In order to test the effect of Sorafenib, it was added as additional input to the system as well as optional edges to possible targets: Raf, EGFR, and IGFR. Note that EGFR as Sorafenib target is a hypothesis and not based on experimental data. Moreover, one cell line, MZ1851RC carries a mutation in mTOR with unknown effect for mTORC1, thus the outgoing edge to IGFR was set to optional. A full list of optional edges in given in Figure 4D, also for some components the logical function can be set, since they only have one regulator (Figure 4C). All other components have undefined logical functions, which gives rise to the generic model pool.

Moreover, components that were neither measured nor perturbed were excluded from the model to reduce the complexity. For example, Mek and Tsc were not considered in the model, since both were lined up in a cascade as components with single input and output, thus deleting them does not pose problems for the model dynamics.

### 3.3. Data formalization

#### 3.3.1. Experiments show differential behavior of cell lines

For our investigation, we used two different data sets: Western blot measurements of mTORC1 activity over time and a high throughput assay both published in Kuznia (2015). In the western blot measurements, the activity of mTORC1 was measured by its targets p70S6K (S6K) and S6RP in MZ1257RC and MZ1851RC cells. Here, the cells were either treated with DMSO or Sorafenib and the phosphorylation of the mTORC1 targets was measured over time. Regarding the measurements until 12 h, MZ1257RC cells showed a significant decrease in phosphorylation levels for S6K and S6RP. However, MZ1851RC cells only showed a reduction in S6RP phosphorylation for later time points, but the phosphorylation of S6K remained high. The 24 h time point is not considered, since we are only interested in signaling effects and this measurement is likely to be influenced by transcriptional effects. S6K was used as the read-out for the mTORC1 activity in the formal encoding of the Western blot data as CTL formulas WB.DMSO, WB1257Sora and WB1851Sora in the Table 1. Here, both cell lines show active mTORC1 for DMSO treatment throughout the measurements, thus a steady state was assumed and encoded in the CTL formula accordingly. For Sorafenib treatment, cell line MZ1257RC shows a steady state with decreased S6K phosphorylation, therefore mTORC1 was set to 0. In contrast, cell line MZ1851RC has stable S6K phosphorylation, thus mTORC1 was set to 1.

**Table 1:** Filtering model pool using model checking.

**CTL formula**	
WB.DMSO:	EF(AG(mTORC1=1)) IS:Sora=0
WB1257Sora:	EF(AG(mTORC1=0)) IS:Sora=1
WB1851Sora:	EF(AG(mTORC1=1)) IS:Sora=1
Bp1851Sora:	EF(mTor=1&Akt=0&EGFR=0&Erk=0&IGFR=1&EF(mTor=1&Akt=1& EGFR=1&Erk=1&IGFR=1&EF(mTor=0&Akt=0&EGFR=0&Erk=0& IGFR=1)))IS:Sora=1
Bp1851DMSO:	EF(mTor=1&Akt=1& EGFR=1&Erk=1&IGFR=1&EF(mTor=1&Akt=1& EGFR=1&Erk=1&IGFR=0&EF(mTor=1&Akt=0&EGFR=1&Erk=1& IGFR=0&EF(mTor=0&Akt=0&EGFR=0&Erk=0&IGFR=0))))IS:Sora=0
Bp1851Sora2:	EF(mTor=1&Akt=1& EGFR=1&Erk=1&IGFR=1&EF(mTor=0&Akt=1& EGFR=1&Erk=1&IGFR=1&EF(mTor=1&Akt=1& EGFR=1&Erk=1&IGFR=0&EF(mTor=1&Akt=0&EGFR=0&Erk=0&IGFR=0&EF(mTor=0&Akt=0&EGFR=1&Erk=1& IGFR=0&EF(mTor=1&Akt=0&EGFR=1&Erk=1&IGFR=1))))))IS:Sora=1
Bp1851DMSO2:	EF(mTor=1&Akt=1& EGFR=1&Erk=1&IGFR=0&EF(mTor=0&Akt=1& EGFR=0&Erk=1&IGFR=0&EF(mTor=0&Akt=0&EGFR=0&Erk=1&IGFR=0&EF(mTor=1&Akt=1& EGFR=1&Erk=1&IGFR=0&EF(mTor=0&Akt=0&EGFR=0&Erk=0&IGFR=0&EF(mTor=0&Akt=0&EGFR=1&Erk=1& IGFR=0&EF(mTor=1&Akt=1& EGFR=1&Erk=1&IGFR=0))))))) IS:Sora=0
Bp1257Sora:	EF(mTor=1&Akt=1&EGFR=1&Erk=1&EF(mTor=0&Akt=0&EGFR=0& Erk=0&EF(mTor=0&Akt=0&EGFR=1&Erk=0&EF(mTor=1&Akt=1& EGFR=1&Erk=1&EF(mTor=0&Akt=0&EGFR=1&Erk=0&EF(mTor=1& Akt=0&EGFR=1&Erk=1))))))IS:Sora=1
Bp1257DMSO:	EF(Delta=0&mTor=1&Akt=1& EGFR=1&Erk=1)IS:Sora=0
Bp1257Sora2:	EF(mTor=0&Akt=0&EGFR=0&Erk=0&EF(mTor=0&Akt=1&EGFR=0& Erk=0&EF(mTor=1&Akt=1&EGFR=1&Erk=1&EF(mTor=1&Akt=1& EGFR=1&Erk=0&EF(mTor=1&Akt=0&EGFR=1&Erk=1&EF(mTor=1& Akt=1&EGFR=1&Erk=1))))))IS:Sora=1
Bp1257DMSO2:	EF(mTor=1&Akt=0&EGFR=1&Erk=1&EF(mTor=1&Akt=0&EGFR=1& Erk=0&EF(mTor=1&Akt=1&EGFR=1&Erk=1&EF(mTor=0&Akt=0& EGFR=0&Erk=0&EF(mTor=1&Akt=1& EGFR=1&Erk=1)))))IS:Sora=0

*CTL formulas derived from Western blot and Bio-Plex® experiments. For denoting the CTL formulas, the following semantics are used: EF(X): is a CTL operator exists finally. This states that on some path from an initial state the X holds true at some point. AG(X): is a CTL operator all globally. This states that X has to hold for all future states, i.e., X is in a steady state. v=b: where v∈V, b∈B states that value of a component v is set to b. IS: declares the initial state and is a list of boolean constraints on the values of the components. A state is considered initial, if all the constraints are satisfied*.

After observing differences in the activity of mTORC1 in the Western blots toward Sorafenib treatment, we wanted to investigate where the differences in the upstream regulation of mTORC1 originate from. For this aim, a high throughput approach using the Bio-Plex® system was applied (Kuznia, 2015). Here, the cells were unstimulated and not starved but treated with Sorafenib or DMSO and measured at different time points over a total period of 36 h in two experiments. In detail, the activity of the PI3K/mTORC1 signaling pathway was measured by the phosphorylation of Akt, and p70S6K as well as the MAPK activity was determined through the phosphorylation of Erk. Moreover, the receptors EGFR, and IGFR were included into the experiment, since we were interested whether the receptors are targeted by Sorafenib and to account for the feedback processes. For the complete dataset see Kuznia (2015), processed data is listed in the Supplementary Table 1.

#### 3.3.2. Discretization of time series data

In order to fit the models in to pool to the time series measurements, the data needs to be discretized. The choice of discretization method is influenced by the kind of data acquired and the experimental method used, for example with large data sets statistical methods provide good results, but with small data sets the choice is more difficult (Dimitrova et al., 2010). Here, we opted to show a simple approach by using the arithmetic mean as threshold. More specifically, the data was discretized by defining for each experiment *e* and component *v*, a threshold

$$\begin{array}{l} \theta_{ev}=\frac{\sum_{t}\sum_{x}m_{evtx}}{\left|t||x\right|} \end{array}$$

where, *m*_*evtx*_ is the measured activity of component *v* in the experiment *e* with treatment *x*. The total number of time points is |*t*| and the total number of treatments is |*x*|.

Since the cells were cultivated and treated in parallel, the phosphorylated levels for both treatments were expected to be comparable. Thus, the threshold for e.g., Erk is the same mean value under both Sorafenib and DMSO treatment within each cell line for each experiment. Moreover, the standard deviation for each component was calculated in order to avoid the problem of discretizing a component that does not change over time. By looking at small standard deviations relative to the mean, IGFR measurements for MZ1257RC in both experiments were identified as problematic (see Supplementary Table 1). Comparing the IGFR levels between the cell lines, we decided to exclude this data.

Since we are interested in the signaling processes, only measurements until 8 h were included. The resulting CTL formulas are listed in Table 1, where all Bioplex measurements were encoded as transient states, due to the fact that they changed throughout the 8 h of measurement. An exception is the data set Bp1257DMSO, which was encoded as steady state (see Supplementary Table 1 MZ1257RC-DMSO Exp1). Note that the discretization of data is not always straight-forward, thus we excluded data which was problematic mathematically (such as IGFR) or had poor quality in the measurements.

##### 3.3.2.1. Robustness of results

As a basic test of robustness with respect to the discretization method being used, we additionally performed a discretization by median instead of mean value. This change in the discretization threshold had a negligible effect on both cell lines: 5.7% of the boolean values changed for MZ1257RC and 7.1% for MZ1851RC. Furthermore, we repeated the subsequent analysis using the median discretization, and observed only minor changes in the size of the model pool for cell line MZ1257RC and no change in the resulting biological interpretation of that pool.

### 3.4. Pool analysis

After deriving the PKN from the literature and including the objectives of the study, the generic model pool was created. As a result from combining of all optional edges and logical equations the pool contains 19,404 models. In order to find biologically relevant models, the third step of the pipeline generated the specific pool(s) by filtering the generic pool for those models that are able to simulate experimentally observed behavior for the two RCC cell lines.

#### 3.4.1. Cell line specific model pools

Each CTL formula has a non-zero pool size and is therefore feasible for our analysis (see Table 2). To determine the cell line specific models, we calculated the intersection of the different subpools for cell line MZ1257RC as Rp.1257 and for cell line MZ1851RC as Rp.1851:

- *Rp*.1851 = *WB*.*DMSO*∩*WB*1851*Sora*∩*Bp*1851*Sora*∩*Bp*1851*Sora*2∩*Bp*1851*DMSO*∩*Bp*1851*DMSO*2
- *Rp*.1257 = *WB*.*DMSO*∩*WB*1257*Sora*∩*Bp*1257*Sora*∩*Bp*1257*Sora*2∩*Bp*1257*DMSO*∩*Bp*1257*DMSO*2

**Table 2:** Number of models consistent with CTL formulas.

**CTL formula**	**Pool size**
**(A)**	
WB.DMSO	15,026
WB1851Sora	5,902
Bp1851Sora	10,080
Bp1851DMSO	12,474
Bp1851Sora2	5,632
Bp1851DMSO2	7,216
Rp.1851	293
**(B)**	
WB.DMSO	15,026
WB1257Sora	15,026
Bp1257Sora	9,984
Bp1257DMSO	12,096
Bp1257Sora2	12,393
Bp1257DMSO2	10,032
Rp.1257	1017

*Filtering for CTL formulas gives pool sizes as the number of models in agreement. Rp.1851 and Rp.1257 are the cell line specific pools as the intersection of the data sets shown in (A,B), respectively*.

Note that both pools are required to fulfill WB.DMSO, since this dataset was identical for both cell lines. Although the single CTL formulas resulted in relatively large pools, containing 5,000–15,000 models, the intersection for the cell line specific pools shows a strong reduction with 1017 models for Rp.1257 and 293 models for Rp.1851 (see Table 2). Thus, there exists a cell line specific pool for each cell line. One interesting question is whether these cell line specific pools share any models, which we addressed by calculating the intersection between Rp.1257 and Rp.1851. The result is an empty set, which means the model pools Rp.1257 and Rp.1851 are disjoint. In the next step, we wanted to further characterize and explore these cell line specific pools for information on crosstalk and Sorafenib targets.

#### 3.4.2. Classification shows differences between cell lines

**Figure 5:**
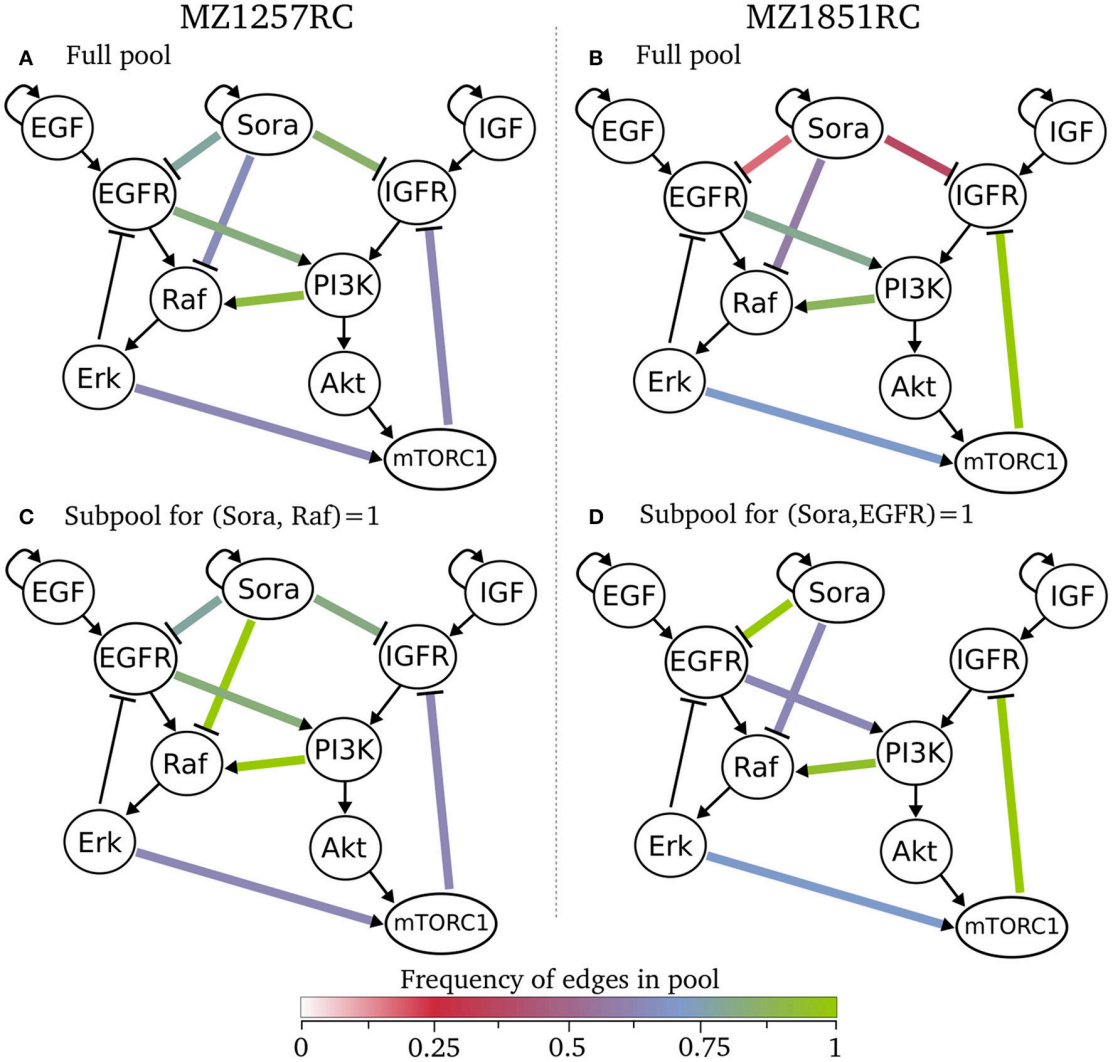
Classification of specific pools for both cell lines, where the color of an optional edge gives the frequency in the respective subpool. In **(A)** all 1017 models for cell line MZ1257RC are visualized and in **(C)** the subpool of 193 models that contain the edge from Sora to Raf is shown. In **(B)** all 293 model of cell line MZ1851RC are presented and in **(D)** a subpool of 72 models with an edge from Sora to EGFR are shown.

Besides the sizes of the pools and the information about the intersection of subpools, we did not receive any information about the models within a pool yet. Since we were interested in the structure of the models, especially the wiring of Sorafenib and crosstalk edges, we selected the classification analysis from the pipeline. Here, we classified for the number of optional edges and the presence of an optional edges. As a result, all models within one class have the same interaction graph, thus only differ in their logical functions (see tables in online repository). Looking at these classes, we can state that for both specific pools there are no rejected edges, since each edge appears in at least one model. This also means that we cannot exclude any of the Sorafenib targets for both cell lines. We can visualize these results by showing the frequency of edges across a pool in Figure 5, where we define the frequency of an edge as the number of models containing this edge divided by the number of models in the pool. Here, the graphs (A) and (B) for the full pools of each cell line show differences in the frequency of the optional edges, especially in the Sorafenib targets and the feedback. While in MZ1257RC the frequency of Sora influences is high, in MZ1851RC they are low especially for IGFR and EGFR. Moreover, one of the objectives was to identify the effect of the mutation in mTOR on the feedback in cell line MZ1851RC, where in Figure 5B 100% of the models in the pool Rp.1857 contain this feedback, while in the other cell line this value only reaches 71%. Also we can observe that every optional edge is present, since no edge is missing.

We can also restrict the classification to a subset of models, e.g., shown in the Figures 5C,D. For cell line MZ1257RC, we selected all models that contain an activating edge from Sorafenib to Raf, where we can observe an enrichment in the crosstalk from PI3K to Raf compared to the full pool (Figure 5C). In contrast, if we filter for all models with an edge from Sora to EGFR in cell line MZ1851RC (Figure 5D), the connection between Sora and IGFR is lost, which means that there is no model containing both edges. Moreover, the frequency of the connection between EGFR and PI3K is reduced. However, since these effects are statistics across a pool of models, it is hard to draw any conclusions about single models.

#### 3.4.3. Minimal mechanisms for sorafenib targets

Although the classification analysis provides a good overview and intuition about the cell line specific pools, the result shows that a more detailed view can provide more information. Looking at the minimal structures or mechanisms of each pool, we wanted to extract more information on how the crosstalk might be linked to the Sorafenib mechanism. For this aim, we analyzed the cell line specific pools for two features: the number of Sorafenib targets and possible crosstalk mechanisms. Due to the large number of models in the pools, we separated the pool for three scenarios: no influence of Sorafenib, meaning that all three optional outgoing edges of Sora are not present, Sorafenib has one target only, Sorafenib has exactly two targets and Sorafenib has exactly three targets. In Table 3, the minimal models according to these scenarios for the pool Rp.1257 and in Table 4 for the pool Rp.1851 are listed.

**Table 3:** Minimal mechanisms for Sorafenib targets and crosstalk in Rp.1257.

	**Sorafenib targets**	**(EGFR, PI3K)**	**(Erk, mTORC1)**	**(mTORC1, IGFR)**	**(PI3K, Raf)**
(A)	None	0	1	0	0
(B)	IGFR	1	0	0	0
		0	1	0	0
	EGFR	1	0	0	0
		0	1	0	0
	Raf	1	0	0	1
		0	1	0	1
(C)	IGFR/EGFR	1	0	0	0
		0	0	1	0
	IGFR/Raf	1	0	0	1
		0	0	1	1
		0	1	0	1
	EGFR/Raf	1	0	0	1
		0	1	0	1
(D)	All	1	0	0	1
		0	0	1	1
		0	1	0	1

*Minimal models after classification of Rp.1257 for (A) no Sorafenib targets, (B) exactly one target, (C) exactly two targets, and (D) all three possible targets are affected*.

**Table 4:** Minimal mechanisms for Sorafenib targets and crosstalk in Rp.1851.

	**Sorafenib targets**	**(EGFR, PI3K)**	**(Erk, mTORC1)**	**(mTORC1, IGFR)**	**(PI3K, Raf)**
(A)	None	0	1	1	0
		1	0	1	0
(B)	IGFR	1	0	1	0
		0	1	1	0
	EGFR	1	0	1	0
		0	1	1	0
	Raf	1	0	1	1
		0	1	1	1
(C)	IGFR/EGFR				
	IGFR/Raf	0	1	1	1
		1	0	1	1
	EGFR/Raf	0	1	1	1
		1	0	1	1
(D)	All				

*Minimal models after classification of Rp.1851 for (A) no Sorafenib targets, (B) exactly one target, (C) exactly two targets, and (D) all three possible targets are affected*.

The specific pool for MZ1257RC shows that every combination of Sorafenib target from none to all is present in the pool, thus we cannot exclude any hypotheses in this cell line (see Table 3). However, we can see that every model contains at least one crosstalk edge and every edge appears in a minimal model. For one Sora target, there is always a basic crosstalk from the MAPK pathway to PI3K signaling either by EGFR on PI3K or by Erk on mTORC1. For Raf, additionally the crosstalk from PI3K on Raf becomes necessary, which we already identified in Figure 5C. For dual targets in Table 3C, IGFR/EGFR requires the cross-activation from EGFR on PI3K or the feedback, IGFR/Raf require (PI3K,Raf) in combination with any of the other crosstalk or the feedback and EGFR/Raf needs (PI3K,Raf) and one crosstalk. In case all three targets are affected by Sora, the (PI3K,Raf) edge and any of the crosstalks or the feedback are required.

The minimal structures in the second cell line MZ1851RC exclude two scenarios: IGFR/EGFR as dual targets (as shown in Figure 5D) and all three targets simultaneously. Table 4 shows models in the pool that are not affected by Sorafenib. Compared to cell line MZ1257RC, there are similarities and difference in the model structures. Raf as a Sorafenib target again requires the edge from PI3K on Raf to be present and also the models always require a crosstalk from the MAPK pathway on PI3K signaling, but additionally the feedback is essential for every model.

#### 3.4.4. Interpretation of analysis results

The minimal models give an overview about how the system could compensate the influence of the inhibitor to fit the data for different levels of influence. For this aspect the cell lines show similarities and differences in their model structures, where two trends can be extracted from the minimal mechanisms. First, all models require at least one crosstalk edge to be able to produce trajectories that match the data we applied. Interestingly, adding more Sorafenib targets most often does not enforce more or different crosstalk edges, with the exception of Raf. Within Rp.1851 the mechanisms for every Sorafenib target and all combinations show the identical minimal mechanism, plus the edge for Raf. A possible explanation for this observation could be the symmetrical structure of the model, in particular when the feedback is active as it is in every model of Rp.1851. Both pathways consist of a cascade of activating edges with a negative feedback on the Sorafenib target. It would be interesting to apply data which breaks with this symmetry, e.g., with a PI3K inhibitor to block the crosstalk.

The second clear trend we can identify from the results is that Raf as Sora target requires the cross-activation from PI3K on Raf. Since Raf is the designated Sorafenib target, this result is interesting. Looking at the PKN structure and the data, we can see that Erk becomes active under Sorafenib treatment and the only activator for Erk is Raf. In the MAPK pathway, Raf is activated by EGFR, which itself is inhibited by Erk. Thus, if Erk should become and stay active over longer time periods as shown in the data, Raf needs another activator to compensate the inhibition through Sorafenib. However, Sorafenib was described to have a paradoxical effect on the MAPK pathway. While, in cell lines carrying a BRAF mutation the signaling was efficiently blocked, cell lines with WT-BRAF showed an activation of Erk (Hatzivassiliou et al., 2010; Heidorn et al., 2010; Poulikakos et al., 2010). Thus, further investigations are necessary to exploit whether this observation is an artifact of the model or has biological relevance. In detail, paradoxical activation by Sorafenib and the role the crosstalk from PI3K to Raf would need to be examined, which would require a refined model where the edge from Sorafenib on Raf could also be activating and more data, e.g., an experiment with a PI3K inhibitor would be interesting in this context.

##### 3.4.4.1. Overlap of sorafenib targets

Another general question is, whether we assume Sorafenib to have the same targets in both cell lines. One could argue that the cell lines could differ in their internal wiring meaning the crosstalk, but the biochemical targets of Sorafenib should be independent of cell lines. Assuming that all three targets, IGFR, EGFR, and Raf, are expressed in both cell lines, the intersection of the results in Tables 3, 4 would further narrow down possible targets. In that case, we could exclude the case of Sorafenib affecting all targets simultaneously, since in cell line MZ1851RC there are no models that have IGFR, EGFR, and Raf as targets. Moreover, the combination IGFR/EGFR is not present in Rp.1851, thus either Sorafenib targets either one of the receptors by themselves or additionally Raf in these cell lines. Even though these results are not clear, they can support and guide further studies, especially experiments where receptors are stimulated additionally to the drug treatment would be beneficial.

##### 3.4.4.2. Models without sorafenib targets

A surprising result of the analysis is the presence of models without a Sorafenib target. In cell line MZ1257RC, < 1% of the models have no Sorafenib target, while the pool for MZ1851RC 16% of the models fall into this category. Since the data clearly shows an effect of the drug on components in this pathway, we expected all models to have at least one target of Sorafenib to be influenced. Thus, the data set from cell line MZ1851RC seems to be not restrictive enough for every model to require an interaction from Sorafenib. Since only a subset of components is measured, some models can match the data by specific initial states. Here, additional data would be beneficial to refine the results, especially measuring more components would reduce the degree of freedom for fitting the data.

## 4. Discussion

In this paper, we present an alternative approach to standard modeling procedures. Instead of building and validating one model, we incorporate uncertain information or hypotheses to build a pool of models that is then filtered for data and analyzed using specific strategies. An advantage of this method is that we can test multiple hypotheses at the same time, but it comes at the cost of high complexity and challenging analysis. For this reason, we created a pipeline with specifically defined objectives and analysis templates that the modeler can select and combine. In addition to templates for objectives, data formalization and pool analysis presented in previous work (Thobe et al., 2014, 2017; Streck et al., 2015), we introduce two new objectives, namely finding driver mutations and drug testing, as well as two analysis options, namely classification and minimal models.

In the second part of the paper, the pipeline is applied to study the uncertain wiring and effect of a drug in cancer cells based on a rich data set. Two RCC cell lines, MZ1257RC and MZ1851RC, were observed to behave differently upon Sorafenib treatment, thus we tested possible drug targets in the MAPK and PI3K signaling and also investigated possible crosstalk between these pathways in a cell line specific manner, incorporating a mutation with uncertain effect as objectives. As a result, a substantial reduction from 19,404 for the initial pool to 1,017 for MZ1257RC and 293 for MZ1851RC was observable, and the empty intersection of both pools shows that the cell line specific models indeed have a different wiring. In order to cope with the complexity of having hundreds of models as outcome of the study, we developed different analysis tools. Here, we showed that classification of the pool can provide an overview on the models in the pool and give information on essential or neglected edges. In the case study, the classification showed that the feedback from mTORC1 on IGFR was active in both cell lines. We had set this edge to optional since the cell line MZ1851RC carries a mutation in mTOR and we hypothesized that this affects the feedback. As a result, all models in Rp.1851 show the feedback in their models and thus the mutation does not affect the function of mTORC1 toward IGFR. However, for cell line MZ1257RC, which does not carry a mutation in mTOR, only 71% of the models in the pool contain this edge. An explanation for this could be that we had to exclude the data for IGFR in the Bioplex experiment, since the variance of the data was too low to allow for a meaningful discretization, which could also be the reason for the larger model pool in comparison to MZ1851RC.

For the crosstalk and the Sorafenib mode of action the classification analysis showed no clear trend, since the results are complex and hard to interpret. For this reason, we listed the minimal models according to the number of Sorafenib targets and the required crosstalk to gain more detailed information on the simplest solution (Tables 3, 4). Even though we cannot exclude any Sorafenib target and crosstalk in the analysis, we are able to identify patterns, where specific Sorafenib targets require different crosstalk edges to be present, e.g., Raf requires a crosstalk from PI3K on Raf. Another important observation from the classification was that there are models in the pools of both cell lines without any interaction between Sorafenib and its target. The conclusion from this result is that the data was not restrictive enough to exclude these models and further data is necessary resolve this issue. However, in case we would only fit one model to the data, we would have missed this lack of expressiveness.

The strength of underlying approach is based on its paradigm of considering possibly huge sets of models for testing and comparison. Consequently, it does not scale as well as single model approaches. The software utilized here is limited by its model checking tool NuSMV, where more than 50 components are not solvable within reasonable time. For the analysis presented in this paper, the program was run on a Ubuntu 17.10 workstation with a processor i7-7700, 3.6 GHz, and 32GB RAM. The script with 10 components took 143 minutes in TomClass, which included building the pool, model-checking, and classification of 19,404 models. Tools like caspo list running times of approximately 56 mins for models of size 45 generating a model pool with 384 models and thus can still handle medium sized models (Videla et al., 2017). The software Tremppi was shown to be able to handle a model pool of size 259,200 and perform model-checking of 40 data sets within 151–177 min depending on parameter settings on a similar workstation (Streck et al., 2016). In general, the kind of models that are feasible for this approach are a trade-off between number of components and number of uncertain edges, the latter of which affects the size of the model pool. This approach aims at exploring uncertainties in small to medium sized models, which is well-suited to represent interesting processes such as signaling pathways and regulatory modules.

While the generation and especially model-checking process is computationally expensive, the analysis and interpretation of these pools, which in our case are just large tables, is challenging from a biological perspective. Thus we propose to define clear objectives for designing the study as well as offer different analysis strategies to extract new information from the complex results. There are many possibilities for extensions especially one could think of further biologically interesting objectives, but also including different kind of data and more analysis options such as algorithms to find special patterns. Moreover, we are not limited to the Boolean set-up, but are able to handle multivalued models (Streck et al., 2015). Finally, although the pipeline was developed for signaling networks, the approach can be applied to any related modeling problem.

## Data and software availability

Python scripts, data sets and the classification of the model pools from the case study analysis are available on GitHub: https://github.com/kthobe/RCC_ModelPoolAnalysis.

The software TomClass is also available on GitHub: https://github.com/hklarner/TomClass.

## Author contributions

KT designed study, analyzed and interpreted data, implemented computations, and drafted the manuscript. CK designed and performed experiments, processed and interpreted data, revised the manuscript. CS participated in discussion, interpreted data, and revised the manuscript. HS participated in conceptualization and discussion, interpreted data, reviewed and revised manuscript. All authors read and approved the final manuscript.

### Conflict of interest statement

The authors declare that the research was conducted in the absence of any commercial or financial relationships that could be construed as a potential conflict of interest.

## Supplementary material

Supplementary Table 1 Dataset from two Bioplex experiments. Each experiment was done for each cell line, where MZ1851RC is shown in the top table and MZ1257RC in the bottom. Both cell lines were treated with DMSO or Sorafenib at the indicated time before measurement and all measured at once, thus one experiment can be seen as one batch. Measurements marked in red were excluded from the analysis due to low variance.
