# Supplementary material for: Evaluating Uncertainty in Signaling Networks Using Logical Modeling
Source: Front Physiol. 2018 Oct 9;9:1335. doi: 10.3389/fphys.2018.01335 (PMC6191669; doi:10.3389/fphys.2018.01335)
Supplement: Supplementary Table 1 — Dataset from two Bioplex experiments. Each experiment was done for each cell line, where MZ1851RC is shown in the top table and MZ1257RC in the bottom. Both cell lines were treated with DMSO or Sorafenib at the indicated time before measurement and all measured at once, thus one experiment can be seen as one batch. Measurements marked in red were excluded from the analysis due to low variance. [file Data_Sheet_1.PDF]

	MZ1851RC-Sora1					MZ1851RC-DMSO1					MZ1851RC-Sora2					MZ1851RC-DMSO2				
	Erk 1/2	EGFR	p70	Akt	IGF-1R	Erk 1/2	EGFR	p70	Akt	IGF-1R	Erk 1/2	EGFR	p70	Akt	IGF-1R	Erk 1/2	EGFR	p70	Akt	IGF-1R
20m	307.5	118	230	540.5	176	737	223	498.5	2468.5	221	629	170	370.5	2133	140	714	175	359	2441	99
40m	460.5	169	344	1941	210	664	223	449.5	2572	227.5	577	167	305	2218	116	573	139	284	2030.5	98
1h	518	200	386.5	2504	247	601	161	328	1850.5	178	567	186.5	323.5	2012	111	531	164	288	1782	84
1,5h	452	160	327.5	2289	195.5	508	202	329	1658	202	545	199	330	1962	103	615	194.5	357.5	1845	86
2h	455.5	146	319	1861	223	439	156	296	1552	162	439	157.5	327	1588.5	106	467.5	157	299	1422.5	96
4h	255	101	205	820	181.5	350	127	236	874	135	535.5	189.5	304	1402	82	591	176.5	297	1571	79.5
8h	232	84	161	517.5	181	221.5	102.5	185	476	137	801	189	366.5	1728.5	171	552.5	188	346	2438	91
12h	200.5	70	152	623.5	139	217	91.5	184	600	117	739	219	400	1564	169.5	425	159	317.5	1773	77
24h	22	42	16	39	59	84	60	80	879.5	90	472	147.5	266	2005	205.5	454	163	335	2490	114
36h	23.5	40.5	15	38	52	61	53	72	286	125	223	101	185	1296	223	240	132	231	1508	100
36h un	65	48	70	200	165	65	48	70	200	165	258	130	245	1546.5	101	258	130	245	1546.5	101
Mean	315	119	225	1127	163	315	119	225	1127	163	509	165	308	1832	116	509	165	308	1832	116
Std	217	61	139	873	53	217	61	139	873	53	158	28	52	355	41	158	28	52	355	41
%	69	51	62	77	32	69	51	62	77	32	31	17	17	19	35	31	17	17	19	35
20m	1	0	0	0	0	1	0	0	0	0	1	0	0	0	0	1	0	0	0	0
40m	1	1	1	1	1	1	1	1	1	1	1	1	1	1	1	1	1	1	1	1
1h	0	0	1	0	1	1	1	1	1	1	1	1	1	1	1	1	1	1	1	0
1,5h	1	1	1	1	1	1	1	1	1	1	1	1	0	1	0	1	0	0	1	0
2h	1	1	1	1	1	1	1	1	1	1	1	1	1	1	0	1	0	0	0	0
4h	1	1	1	1	1	1	1	1	1	1	1	1	1	1	0	1	1	1	1	0
8h	1	1	1	1	1	1	1	1	1	0	0	0	1	0	0	0	0	0	0	0

	MZ1257RC-Sora1					MZ1257RC-DMSO1					MZ1257RC-Sora2					MZ1257RC-DMSO2				
	Erk 1/2	EGFR	p70	Akt	IGF-1R	Erk 1/2	EGFR	p70	Akt	IGF-1R	Erk 1/2	EGFR	p70	Akt	IGF-1R	Erk 1/2	EGFR	p70	Akt	IGF-1R
20m	353.5	136	278	569	48	491	178.5	355	659	58	298	103	228	422	50	358	121	257	442.5	47
40m	157	81	149	444.5	49	383.5	143.5	305	787	51	301	115	241.5	876.5	47	350.5	125.5	267	584	42
1h	160.5	83	142	365	44	386	138.5	308	665	49	489	154	357	1006.5	40	464	122.5	303	834	42
1,5h	204	128	231	430	50	387	155	350	817.5	48	346	131	290	735	37	314	108	204	653	35
2h	253	160	290	557	46	379	152.5	357.5	847	51	366	120	293	694.5	41	339.5	121	232	542	36
4h	196	139	205	411	44	446.5	188.5	424	973	49	476	135	323	721	36	524	136	294	748.5	35
8h	288.5	224	335	434	55	346	153.5	313	468	56	508	154.5	351	1086	50	484	141.5	295	927	41
12h	67	67	94.5	115	51	264	137.5	279	449.5	48	390	128	279	854	45	537	157	402	999	38
24h	73	67	84	345.5	53	168	103	176	411	44	227	95	171	679	42	439	136	314	945	36
36h	39.5	56	49	82	48	156	127	214.5	169.5	42	63	48	80	302.5	33	131	85	166.5	455	33
36h un	92	54	117.5	86.5	45	92	54	117.5	86.5	45	185	91.5	164	364	31	185	91.5	164	364	31
Mean	245	124	235	462	49	245	124	235	462	49	353	119	258	693	39	353	119	258	693	39
Std	136	47	106	259	4	136	47	106	259	4	133	26	77	234	6	133	26	77	234	6
%	55	38	45	56	9	55	38	45	56	9	38	22	30	34	15	38	22	30	34	15
20m	1	0	0	0		1	0	0	0		1	0	0	0		1	0	0	0	
40m	1	1	1	1		1	1	1	1		1	1	1	1		1	1	1	1	
1h	1	1	1	0		1	1	1	0		0	0	0	0		0	0	0	0	
1,5h	0	0	0	0		1	1	1	0		0	0	0	0		0	0	0	0	
2h	0	0	0	0		1	1	1	0		0	0	1	0		0	0	0	0	
4h	0	1	1	0		1	1	1	0		0	0	0	0		0	0	0	0	
8h	0	1	1	0		1	1	1	0		0	0	0	0		0	0	0	0	
